# Gastric Artery Injury Due to Blunt Abdominal Trauma

**DOI:** 10.7759/cureus.50018

**Published:** 2023-12-06

**Authors:** Saaya Ichiyama, Yoshiya Ishizawa, Keisuke Washida, Shinya Kakehata, Shingo Kakeda

**Affiliations:** 1 Emergency and Disaster Medicine, Hirosaki University, Hiorosaki, JPN; 2 Emergency and Critical Care Center, Aomori Prefectural Central Hospital, Aomori, JPN; 3 Radiology, Hirosaki University, Hirosaki, JPN

**Keywords:** interventional radiology-guided embolization, gastric artery, blunt abdomen trauma, selective non-operative management, airbag-associated injury, ruptured pseudoaneurysm

## Abstract

Gastric artery injury resulting from blunt abdominal trauma is rare, with only eight previous cases documented in the published literature. Our report describes a case involving an injury to the right gastric artery with concomitant injuries to the liver and spleen, for which arterial embolization targeting the right gastric artery was performed.

The patient, a 66-year-old woman without any remarkable medical history, was involved in a motor vehicle accident. She was brought to the hospital in a state of shock and complaining of upper abdominal pain. Contrast-enhanced CT indicated hepatic and splenic injuries, intra-abdominal hemorrhaging, and effusion of contrast medium, suggesting involvement of the right gastric artery.

Subsequent angiography confirmed irregularities in the diameter of the right gastric artery, prompting coil embolization. A conservative therapeutic approach was selected due to the absence of evidence regarding active hemorrhage or vascular injury within the hepatic or splenic regions. The patient remained clinically stable following the embolization, without any sequelae.

Arterial embolization is warranted if preoperative contrast CT indicates signs of hemorrhage, even if hemostasis is ostensibly attained during angiography. Our findings allude to the feasibility of non-operative management (NOM) rather than laparotomy for cases of gastric artery injury.

## Introduction

Gastric artery injury resulting from blunt abdominal trauma occurs infrequently, with a total of only eight cases reported in published literature. These encompass five cases of pseudoaneurysms [1-5] and three cases of active hemorrhage [6-8]. Notably, all instances of active hemorrhage were managed through laparotomy and the subsequent ligation of the gastric artery [6-8]. Herein, we present a case of successfully treated arterial embolization of the right gastric artery without any sequelae, accompanied by an in-depth review of the pertinent literature.

## Case presentation

**Figure 1:**
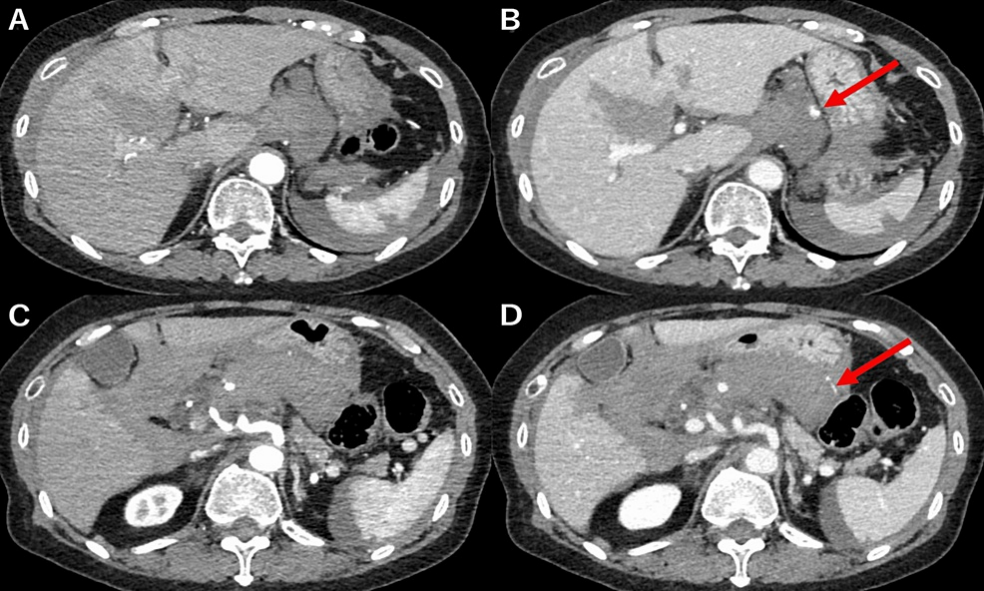
Contrast-enhanced computed tomography Contrast-enhanced computed tomography was conducted at the time of injury, with panes A and C showing the arterial phase and panes B and D the venous phase. The scan shows hepatic injury, splenic injury, and intra-abdominal hemorrhage, along with the presence of extravascular leakage of the contrast medium (red arrow).

The patient, a 66-year-old woman with no remarkable medical history, was operating a lightweight vehicle at a speed of 50 km/h when she accidentally broadsided an oncoming vehicle. Emergency medical personnel noted she was in a state of shock, and she also complained of upper abdominal pain. She was brought to a nearby hospital under suspicion of abdominal organ damage. Upon arrival, her upper abdominal pain persisted, but she maintained clarity of consciousness. Assessment of her vital signs revealed a respiratory rate of 28 breaths/min, oxygen saturation (SpO_2_) of 97% in ambient air, systolic blood pressure oscillating in the 70 mmHg range, and a heart rate of 79 beats per minute. The patient also noted peripheral cold sensations. Following the administration of extracellular fluid and four units of irradiation of the red blood cells, her systolic blood pressure stabilized to approximately 100 mmHg. Notably, the focused assessment with sonography for trauma (FAST) yielded negative results, and radiographic examinations of the chest and pelvis did not show any aberrations. The patient complained of spontaneous pain and tenderness in the upper abdominal region. Subsequent simple and contrast-enhanced CT from the head to pelvis showed a left fourth to eighth rib fracture, coupled with liver injury (grade IIIa according to the Japanese Association of Traumatology Classification of Liver Injury 2008/grade II according to the American Association for the Surgery of Trauma (AAST)) and splenic injury (grade IIIa according to the Japanese Association of Traumatology Classification of Splenic Injury 2008/grade II according to the AAST). The patient was subsequently transferred to our institution due to suspected right gastric artery involvement as the source of the bleeding, which was substantiated as intra-abdominal hemorrhage and extravascular leakage of contrast medium observed during the equilibrium phase on contrast-enhanced CT (Figures 1A-1D).

**Figure 2:**
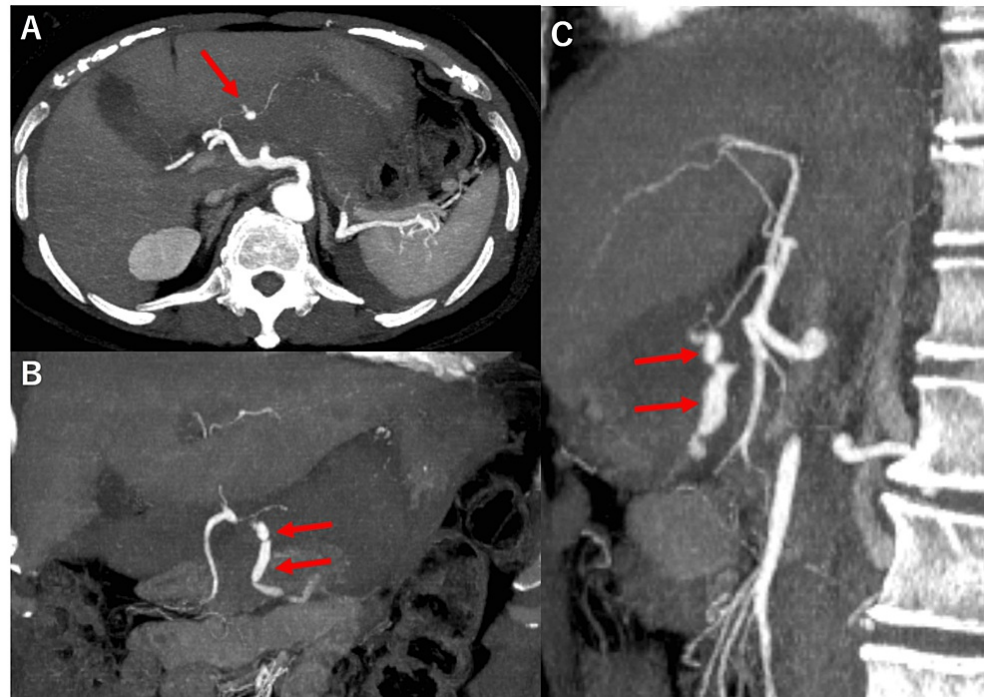
Reconstructed contrast-enhanced computed tomography Reconstructed contrast-enhanced CT was performed at the time of injury, with pane A showing the axial, pane B the coronal, and pane C the sagittal images. Since there was a pseudoaneurysm in the right gastric artery and continuous extravasation (red arrow), it was judged that the right gastric artery was the injured vessel.

Upon admission to our hospital, the patient's systolic blood pressure had increased to the 110 mmHg range, indicating amelioration of her shock condition. Subsequent to the initial stabilization of the patient's hemodynamics, a review of the contrast-enhanced CT conducted at the preceding medical facility confirmed the presence of a right gastric artery injury (Figures 2A-2C), prompting the decision to perform arterial embolization.

**Figure 3:**
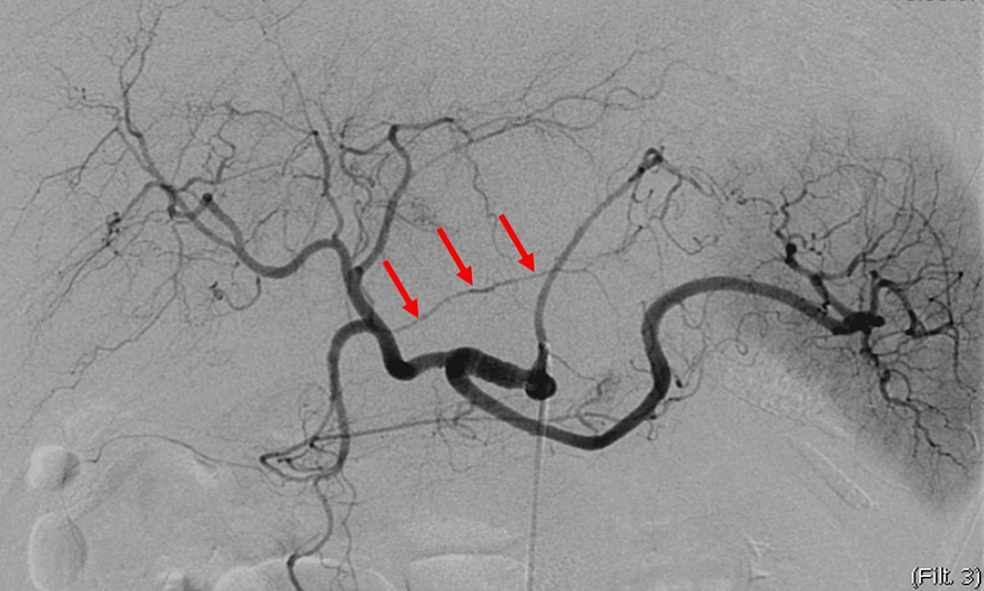
Celiac artery angiography There are no signs of an aneurysm or active hemorrhage. However, irregularities in the diameter of the right gastric artery are apparent.

A long sheath was introduced into the right femoral artery, facilitating angiography of the celiac artery using a Shepherd hook catheter. Angiography findings revealed irregularities in the diameter of the right gastric artery (Figure 3).

While the angiogram did not reveal any aneurysms or active bleeding (Figure 3), coil embolization of the right gastric artery was performed due to vessel irregularities and compelling indications of structural damage, along with the potential for rebleeding or pseudoaneurysm formation.

**Figure 4:**
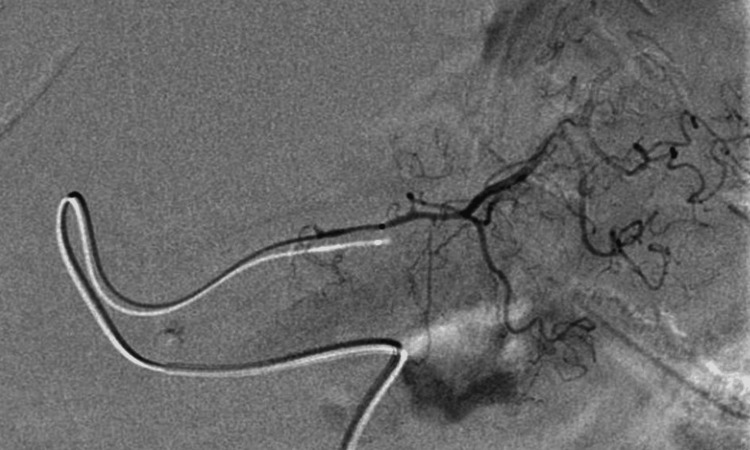
Right gastric artery angiography The right gastric artery is selectively contrast-enhanced, but there is no evidence of active bleeding.

Subsequent angiography confirmed the complete embolization of the afflicted vessel (Figure 4).

**Figure 5:**
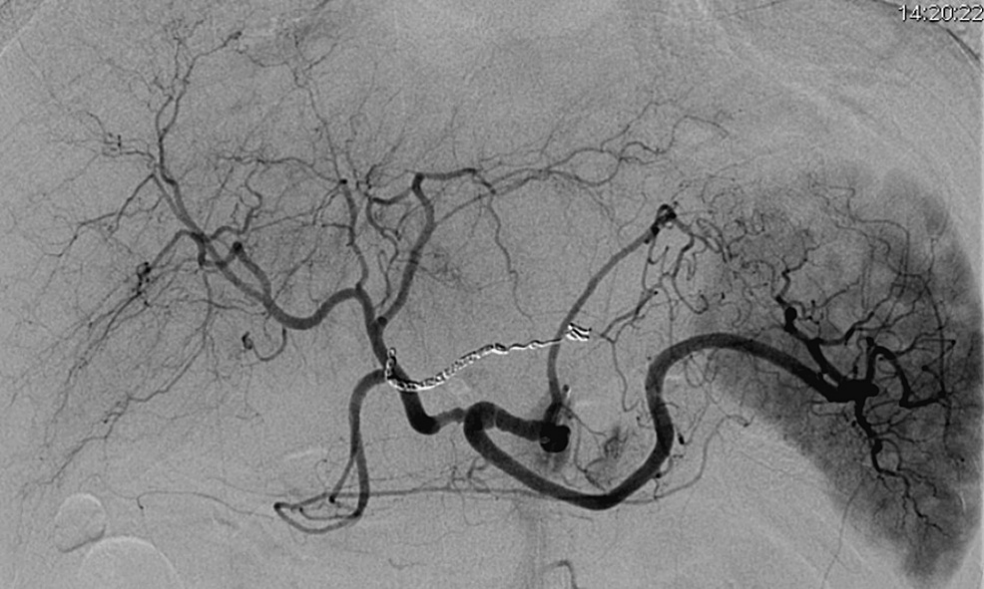
Angiography performed after the coil embolization of the right gastric artery The right gastric artery has been embolized completely.

No additional arterial injury was suspected due to the absence of any signs of active bleeding, pseudoaneurysm formation, or irregular diameter within the hepatic artery, splenic artery, or left gastric artery. Consequently, further embolization was deemed unwarranted, and a conservative treatment approach was adopted.

Post-embolization, the patient's cardiopulmonary status remained stable. However, her fibrinogen levels decreased to 157 mg/dL, necessitating the transfusion of eight units of fresh frozen plasma. On the second day following the injury, contrast-enhanced CT confirmed the resolution of extravascular contrast leakage, the absence of pseudoaneurysms, and a reduction in the volume of hematoma. On the fifth day after the injury, the patient was transferred to her initial healthcare provider, and contrast-enhanced CT performed on the sixth day after the injury did not reveal any indications of rebleeding, pseudoaneurysm development, or abscess formation. On the seventh day following the injury, the patient was discharged to her residence, where she currently receives outpatient care. Remarkably, she has resumed her daily activities without any sequelae.

## Discussion

The patient in the present case suffered damage to her right gastric artery, concurrent with liver and splenic injuries resulting from blunt abdominal trauma. Angiography did not reveal any extravascular contrast leakage; however, the right gastric artery was deemed compromised due to irregularities in its diameter. This prompted the implementation of coil embolization of the right gastric artery and the adoption of non-operative management (NOM).

It is estimated that vascular injuries constitute approximately 3% of all traumatic incidents [9], and gastric artery injuries resulting from blunt abdominal trauma are rare. To the best of our knowledge, there are only eight previous reports, encompassing five cases of pseudoaneurysms [1-5] and three cases of active hemorrhage [6-8]. The direct mechanism of vascular injury was reported to be the result of rapid compression and drainage of abdominal organs attached to the abdominal wall with the great omentum from the impact of an external force on the upper abdomen [7]. These clinical manifestations, such as abrasion, subcutaneous hematoma, or intramuscular hematoma of the abdominal wall, negate the likelihood of an abdominal wall injury, thus making handlebar trauma improbable. Abdominal trauma associated with the deployment of an airbag occurs in two distinct phases: the initial impact upon release and the consequent pressure surge during inflation [10]. When the airbag deploys while the patient is securely fastened with a seatbelt, the combination of belt pressure and airbag inflation elevates intra-abdominal pressure, thereby increasing the likelihood of injuries to the liver and spleen [11]. In the context of the present case, it is postulated that pressure due to airbag activation elevated the patient's intra-abdominal pressure, inducing a compressive force on the abdomen along the anterior-posterior axis. This, in turn, may have resulted in hyperextension of the right gastric artery, potentially precipitating injury. It is also possible that the patient was not wearing a seatbelt, and the airbag's deployment overlapped the gastric artery, culminating in a gastric artery injury.

In cases of blunt hepatic injury, NOM is indicated when hemodynamics are stable, there is no other trauma requiring surgery [12], and the treatment success rate is more than 80% [11]. For blunt splenic injuries, NOM is indicated when hemodynamics are stable [13], and the treatment success rate for NOM using arterial embolization in cases of traumatic splenic injury of AAST grade III or higher is more than 80% [14]. In this case, liver and splenic injuries were indicated for NOM. There is no established treatment for a gastric artery injury. In previous reports, all patients with active bleeding underwent laparotomy and had their gastric artery ligated [6-8]. In the present case, emergency laparotomy was indicated when the patient's hemodynamic status was unstable despite initial treatment; however, the patient's shock improved after the transfusion of fluids and blood. Moreover, contrast-enhanced CT conducted at the previous hospital indicated that the right gastric artery was the responsible vessel, so we decided to perform arterial embolization. Angiography showed no aneurysm or extravasation of contrast medium in the right gastric artery, but the diameter of the vessel was irregular, and damage was highly suspected. Therefore, we embolized it in consideration of the possibility of rebleeding and pseudoaneurysm. In a review of previous reports of gastric artery injuries, pseudoaneurysms were found in five out of eight cases. All of the cases with active bleeding or pseudoaneurysms with active bleeding were diagnosed and treated on the same day of injury, while two cases with only pseudoaneurysms were reported within one week of injury and one case with a delayed onset was reported within one month after injury (Table 1).

**Table 1:** A summary of the eight previously reported cases of gastric artery injury due to blunt abdominal trauma

Authors	Age, sex	Mechanism of injury	Injured vessel	Form of damage	Time from injury to diagnosis	Associated injuries	Treatment
Varela JE et al., 2006 [1]	43 years, male	Head-on motor vehicle collision	Left gastric artery	Pseudoaneurysm with acute extravasation	Same day	Left hepatic lobe, left seventh and eighth rib fractures, L5 right transverse process fracture, left acetabular fracture	Embolization
Mathew AJ et al., 2007 [2]	24 years, male	Blunt abdominal trauma	Left gastric artery	Pseudoaneurysm	34 days	None	Embolization
Allorto NL et al., 2009 [3]	1 year, male	Blunt abdominal trauma	Left gastric artery	Pseudoaneurysm	7 days	None	Embolization
Noh D et al., 2019 [4]	79 years, male	Slipping and falling down	Left gastric artery	Pseudoaneurysm	2 days	None	Embolization
Nissim L et al., 2017 [5]	25 years, male	Head-on motor vehicle collision	Left gastric artery	Pseudoaneurysm with acute extravasation	Same day	None noted	Embolization
Udekwu OP et al., 1993 [6]	26 years, male	Head-on collision	Right gastric artery, gastroduodenal artery	Acute extravasation	Same day	Left medial malleolar ankle fracture	Ligation
Nagata H et al., 2005 [7]	54 years, male	Handlebars of an overturned truck struck the abdomen	Right gastric artery	Acute extravasation	Same day	Fasciae laceration of rectus abdominis muscle with disposition of some greater omentum out of the abdominal cavity to the subcutis	Ligation
Yunoki Y et al., 2001 [8]	58 years, male	Side mirrors of trucks collided with each other	Left gastric artery	Acute extravasation	Same day	None	Ligation

Even in instances such as the present case, where hemostasis was achieved at the time of angiography, if there is active bleeding on preoperative contrast-enhanced CT, arterial embolization is recommended because there is a high possibility of delayed formation of a pseudoaneurysm and rebleeding. In addition, the results of this study suggest that NOM may be indicated in cases in which shock has improved after initial treatment and the patient's hemodynamic status remains stable.

## Conclusions

We encountered a case of gastric artery injury resulting from blunt abdominal trauma. Preoperative contrast-enhanced CT showed evidence of active hemorrhage, implying that arterial embolization yields a favorable prognosis, even without aneurysmal pathology or active bleeding upon angiographic assessment. Despite reports of previous cases managed via laparotomy, our findings advocate for the feasibility of NOM in cases that exhibit sustained hemodynamic stability following initial interventions.
